# Recent Advances in Materials and Flexible Sensors for Arrhythmia Detection

**DOI:** 10.3390/ma15030724

**Published:** 2022-01-18

**Authors:** Matthew Guess, Nathan Zavanelli, Woon-Hong Yeo

**Affiliations:** 1 George W. Woodruff School of Mechanical Engineering, Georgia Institute of Technology, Atlanta, GA 30332, USA; mguess7@gatech.edu (M.G.); nzavanelli@gatech.edu (N.Z.); 2 Center for Human-Centric Interfaces and Engineering, Institute for Electronics and Nanotechnology, Georgia Institute of Technology, Atlanta, GA 30332, USA; 3 Wallace H. Coulter Department of Biomedical Engineering, Georgia Institute of Technology, Atlanta, GA 30332, USA; 4 Parker H. Petit Institute for Bioengineering and Biosciences, Neural Engineering Center, Institute for Materials, Institute for Robotics and Intelligent Machines, Georgia Institute of Technology, Atlanta, GA 30332, USA

**Keywords:** arrhythmia detection, cardiovascular monitoring, soft biosensors, wearable sensors, flexible electronics

## Abstract

Arrhythmias are one of the leading causes of death in the United States, and their early detection is essential for patient wellness. However, traditional arrhythmia diagnosis by expert evaluation from intermittent clinical examinations is time-consuming and often lacks quantitative data. Modern wearable sensors and machine learning algorithms have attempted to alleviate this problem by providing continuous monitoring and real-time arrhythmia detection. However, current devices are still largely limited by the fundamental mismatch between skin and sensor, giving way to motion artifacts. Additionally, the desirable qualities of flexibility, robustness, breathability, adhesiveness, stretchability, and durability cannot all be met at once. Flexible sensors have improved upon the current clinical arrhythmia detection methods by following the topography of skin and reducing the natural interface mismatch between cardiac monitoring sensors and human skin. Flexible bioelectric, optoelectronic, ultrasonic, and mechanoelectrical sensors have been demonstrated to provide essential information about heart-rate variability, which is crucial in detecting and classifying arrhythmias. In this review, we analyze the current trends in flexible wearable sensors for cardiac monitoring and the efficacy of these devices for arrhythmia detection.

## 1. Introduction

Arrhythmia is the presence of abnormal cardiac rhythms. In 2018, more than 500,000 American deaths included arrhythmia as a contributing factor, demonstrating its deleterious impact on patient health [1]. Furthermore, the lifetime risk of atrial fibrillation in the United States is estimated to be one in three among Caucasians and one in five among African Americans [2]. Arrhythmias occur when the electrical pulses of the heart are not functioning properly, causing the heart to beat either too fast, too slow, or skip beats. Impulse-production arrhythmias can be grouped into six categories: premature beats, non-sinus rhythm, fibrillation, tachycardias, bradycardias, and flutter. Premature beats are abnormally timed beats that occur before the sinus rhythm and are caused by the heart being unable to fill with the appropriate amount of blood [3]. Atrial fibrillation, the most common arrhythmia, occurs when the electrical pulses between the upper chambers of the heart, the atria, do not sync with the pulses in the lower chambers of the heart, the ventricles. Ventricular fibrillation, on the other hand, occurs when there is a mismatch between the right and left atria, which makes the heart unable to pump blood to the body [3]. Tachycardias occur when the heart is beating too fast, generally more than 100 beats per minute, and bradycardias occur when the heart is beating too slow, generally less than 40 beats per minute. In addition, impulse-conduction arrhythmia types include atrioventricular block, bundle branch block, Wolff—Parkinson—White syndrome, and escape beats [4]. An atrioventricular block occurs when the impulses between the atria and ventricles become blocked due to a failure in the heart’s conduction system. Bundle branch block occurs as a result of blockages in the pathways in the heart. Wolff—Parkinson—White syndrome occurs when additional electrical pathways are made between the atria and ventricles, resulting in a rapid heartbeat [5].

Despite the clear need for early arrythmia detection to avoid these serious complications, existing detection mechanisms have proven insufficient. Arrhythmias have traditionally been diagnosed by medical professionals based on qualitative data, a patient’s medical history, and clinical examinations. Electrocardiography (ECG) has proven instrumental in identifying arrhythmias. The importance of continuous monitoring for specific arrhythmias has been increasingly identified, as both asymptomatic arrhythmias and paroxysmal diseases remain difficult to detect through intermittent clinical ECG recordings [6,7]. The 12-lead Holter monitor has long been the clinical standard for detection and diagnosis of heart-rate diseases using long-term monitoring of ECG [8]. Though these devices are widely used, they are prone to poor patient compliance because of their bulkiness and reliance on wired leads [9]. In addition, these devices experience signal deterioration over time due to the drying of the conductive gels [10]. The advent of miniaturized, one-lead devices has offered an alternative to multi-lead ECG devices. However, they are susceptible to motion artifacts that disrupt data collection [11].

**Figure 1:**
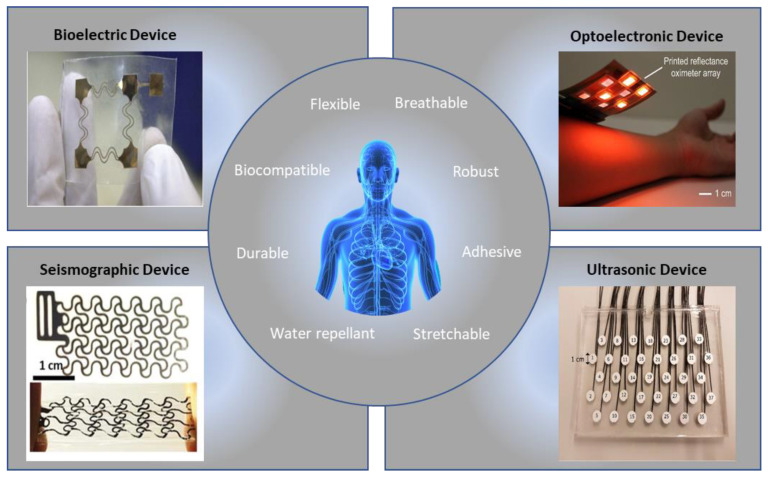
Examples of flexible sensors and functions for accurate arrhythmia detection [13,14,15,16]. (Figures are adapted or reprinted, clockwise, from the top–left: (1) *Sensors Actuators A Phys.* 2018, *272*, 92–101, Copyright 2019, Elsevier; (2) *Proc. Natl. Acad. Sci.* 2018, *115*, E11015–E11024, Copyright 2018, National Academy of Sciences; (3) Creative Common License by MDPI; (4) Creative Common License by Wiley.

Flexible devices have emerged as alternatives to these rigid devices, eliminating motion artifacts by increasing sensor-to-skin adhesion. Recently, new areas of research have been developed to make these heart-rate monitoring devices cheaper and faster to manufacture, expanding accessibility for these previously costly devices. Table 1 shows the currently available flexible devices for arrhythmia monitoring. Additionally, new alternatives to ECG can provide information that ECG alone cannot. For instance, photoplethysmography, ultrasound, seismocardiography, and ballistocardiography can characterize the heart’s electromechanics. Figure 1 shows these soft-sensor types, along with the desirable qualities of the devices. New arrhythmia-detection methodologies also offer more accurate, automatic information to patients for a low cost. For example, deep neural networks, which are capable of learning important features and patterns without extensive preprocessing or feature engineering, are becoming extremely accurate in predicting types of arrhythmia [12].

In this review, we summarize the types of sensors used to detec arrhythmias, with an emphasis on non-implantable devices and recent advances in the flexibility of previously rigid sensor types. Different sensing methods can offer low-cost alternatives to traditional sensing or provide information that is unobtainable with other sensors. We explore the materials needed to fabricate these flexible devices and discuss the mechanical, chemical, and electrical properties of these materials, as well as the effect of these properties on the detection of arrhythmias. Next, arrhythmia detection methodologies of various arrhythmia-detection devices are explored, along with their limitations. Finally, we comment on the current cutting-edge research in the field, the current problems and possible solutions, and the future development of wearable sensors for arrhythmia detection.

## 2. Bioelectric Signals

### 2.1. Mechanics of ECG

ECG is the practice of measuring the heart’s electrical activity using pairs of electrodes on the skin. Clinically, this is achieved with 12 leads using 10 electrodes to measure cardiac signals from many angles [23]. A healthy ECG cycle typically consists of five different waves: P, Q, R, S, and T. The P-wave is the first positive wave and corresponds to atrial depolarization. The QRS complex, which consists of a negative Q-wave, a large positive R-wave, and a negative S-wave, represents ventricular depolarization and ventricular contraction. The T-wave represents ventricular repolarization and ventricular diastole [24]. Distortion of these waves can indicate abnormalities in the heart rhythm [25]. The Pan-Tompkins algorithm is commonly used to find the QRS complex in a regular ECG rhythm [26]. Although 12-lead ECGs provide the best clinical method for diagnosing arrhythmias, the switch to 3-lead ECGs or single-lead ECGs offers many advantages [23]. Cardiovascular patches that use adhesives have become increasingly popular due to their unobtrusiveness. For example, the FDA-approved Zio patch (iRhythm Technologies, Inc., San Francisco, CA, USA) has shown clinical suitability for detecting arrhythmias when compared with a 12-lead Holter monitor over 14 days [27].

### 2.2. Materials for Flexible ECG Devices

**Figure 2:**
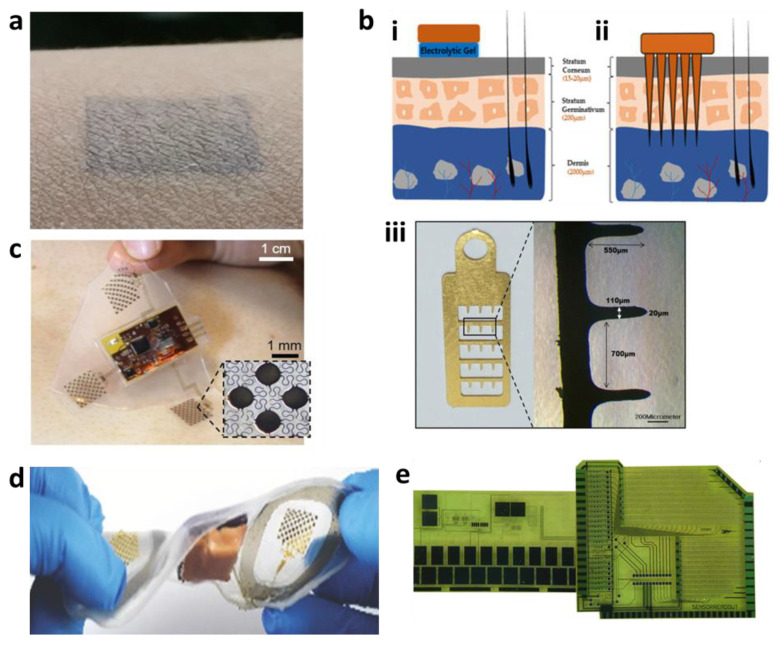
Electrocardiography. (**a**) Photo of a skin-conformal electrode. (**b**) Microneedle array-based ECG. Illustration of (**i**) traditional Ag/AgCl electrode and (**ii**) microneedle array electrode. (**iii**) Photo of a microneedle array electrode. (**c**) Photo of a stretchable hybrid-electronics device. (**d**) Photo of a soft strain-isolated bioelectric device. (**e**) Foil micrograph of a flexible ECG patch.

Many ECG electrodes currently used in clinics are made up of three parts: (1) a conductive metal, traditionally Ag/AgCl electrodes; (2) a conductive gel; and (3) an adhesive patch. Conductive gels reduce the impedance from the electrode to the skin. However, they dry up over time, which causes signal quality to deteriorate during long-term monitoring. Therefore, many materials have been explored as alternatives to traditional Ag/AgCl that are conformal to the skin. Although they generally have higher impedances than wet electrodes, flexible dry electrodes are quickly gaining in popularity. Electrodes can be made from any conductive materials. Since metals have high Young’s moduli, ultra-thin metal films can be arranged in serpentine or fractal geometries to provide flexibility or stretchability, as shown in Figure 2a [28,29,30,31]. Chlaihawi et al. reported an electrode that was screen printed with Ag flake ink [32]. The electrode with the largest area reported a 0.95 correlation coefficient with traditional wet Ag/AgCl electrodes. In addition, the study showed the feasibility of using the high-throughput process of screen printing for the development of flexible dry electrodes.

The stretchability of these thin-film metals can be increased by introducing conductive polymers. Polyethylene terephthalate (PET) and polydimethylsiloxane (PDMS) are commonly chosen as polymers due to their biocompatibility, wide availability, and low Young’s modulus. To make these polymers conductive, materials such as activated carbon or metal micro/nanoparticles are added to form networks of conductivity. For example, Jung et al. showed a carbon nanotube (CNT)/PDMS composite-based dry electrode to combat motion and sweat artifacts [33]. The performance of the electrode was able to be tuned by adjusting the CNT concentration. This electrode showed no signal degradation over a seven-day period of continuous monitoring, providing similar motion-artifact reduction as wet electrodes and more motion-artifact reduction than other dry electrodes. Zhang et al. showed an electrode that had stretchability up to 500% by combining Ag nanowires (NWs) with polymers [34]. This sensor improved upon the CNT/PDMS structure, which is subject to weak connection of conductive materials when stretched. This polymer/Ag NW sensor also increased the durability over 1000 cycles and exhibited good fatigue resistance.

In recent years, the conductive polymer poly (3,4-ethylene dioxthiophene): polystyrene trans acid (PEDOT:PSS) has been the most common polymer for textile-based electrodes due to its high sensitivity to biological molecules and high response time [35]. Wang et al. showed that PEDOT:PSS could be used to achieve even higher flexibility and lower skin impedance by combining it with a flexible cellulose/polyvinyl alcohol substrate [20]. This process provides new ideas for low-cost manufacturing of environmentally friendly ECG devices.

In addition to material advances, recent studies have decreased the impedance of surface electrodes by changing the form factor. For example, semi-invasive strategies, such as microneedle-based approaches, have been demonstrated to reduce motion artifacts [36,37]. Satti et al. reported a microneedle array electrode (MNE), as shown in Figure 2b, that showed no mechanical failure under compression forces of 16 N and showed that while the signal quality of wet Ag and AgCl electrodes decreased after 3 days and 1 week, respectively, the MNE showed no signs of signal-quality deterioration [36].

Despite the many developments in electrode technology, there is still a lack of all-in-one integration. To address these sensor-only systems, innovations in packaging have become important as well. For example, the general impedance challenges of sweat buildup on electrodes have been addressed by integrating hydrophilic poly(urethane-acrylate) into Ag electrodes to increase conductivity during sweating and increasing the breathability of the substrate, as shown in Figure 2c,d [37,38,39,40,41]. All-in-one systems featuring wireless charging, wireless data communication, and onboard data analysis have been developed, as in Figure 2e [42,43,44,45].

## 3. Optoelectronic Signals

### 3.1. Mechanics of PPG

Photoplethysmography (PPG) is emerging as an alternative to ECG for cardiovascular monitoring due to its small size and ability to capture many different physiological parameters. The LED operates at red and near-infrared (NIR) frequencies, and the light intensity reaching the photodiode changes depending on the volumetric changes in the veins and arteries [46]. PPG sensors consist of two basic components: a light-emitting diode (LED) and a photodiode. The PPG sensor can function in two main modes: (1) transmission, where the LED and photodiode are placed on opposite sides of the medium; or (2) reflection, where the LED and photodiode are placed on the same side of the medium [47]. Due to the many factors affecting blood flow, including cardiac, neural, and respiratory factors, it is possible to look at many physiological parameters. PPG is currently used to measure several different aspects of heart health, including blood oxygen saturation (SpO2), blood pressure, heart rate, and respiratory rate [48,49,50,51,52].

Because PPG sensors only require two simple components, an LED and photodiode, they are commonly implemented in already-existing devices, such as watches and smartphones, at low cost. However, the fundamental mismatch between the shape and rigidity of these devices with the skin makes them prone to heavy motion-artifact noise. Thus, flexible materials are essential for increasing the accuracy of optoelectronic sensors.

### 3.2. Materials for Flexible PPG Devices

Both flexible LEDs and flexible diodes have been developed to reduce the effects of motion artifacts, which have been difficult to remove with filtering alone [53]. The most common photodiodes currently in use are silicon photodiodes, as they are widely available and flexible. Kim et al. used flexible (PIN) silicon diodes in combination with near-field communication (NFC) to deliver power, eliminating the need for a battery [54]. The photodiodes were paired with red and infrared LEDs, and the signals were amplified to coils and sent to a smartphone using the NFC platform. Li et al. offered an improvement on the conventional optoelectronic architecture by designing an epidermal silicon-based device by using a specific strain-isolation design, nanodiamond thinning, and hybrid transfer printing [55]. Through the thinning process, the thickness of the LEDs and PD was reduced to 20 μm. Mechanical deformation was addressed by adding a flexible island in a sandwich structure, with PI and PDMS helping the device to show stable operation, even under a strain of 35%. This device promised the possibility for functional optoelectronic devices to be directly mounted on the skin. Gallium arsenide (GaAs) is a frequently used III-V semiconductor material that can be used as an alternative to Si-based materials based on its excellent charge-carrier mobility and high stability. Hong et al. demonstrated a GaAs-based flexible photodetector array that was hetero-epitaxially grown on a Si wafer [56]. This innovative manufacturing method showed promising results that could lower the cost of inorganic photodiodes, which are normally expensive. This platform shows promising possibilities for large-scale creation of flexible photodiodes.

**Figure 3:**
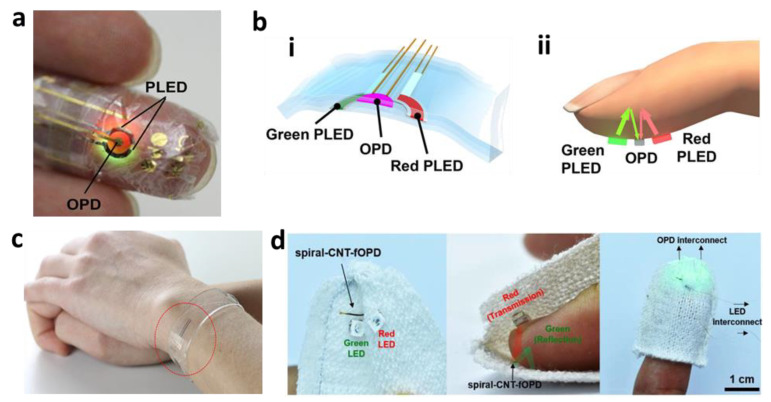
Photoplethysmography. (**a**) Photo of an ultra-flexible organic optical sensor. (**b**) Illustration of polymer LED (**i**) and organic photodiode (**ii**) pulse oximeters. (**c**) Photo of a graphene-based flexible sensor in a heart-rate monitoring bracelet. (**d**) Photos of CNT-based microelectrodes for a fiber optoelectronic device.

Organic materials for PPG sensors have become increasingly attractive due to their low fabrication cost and environmentally friendly footprint. For example, Yokota et al. developed a flexible pulse oximeter consisting of a polymer LED (PLED) and an organic photodiode (OPD) (Figure 3a,b) [57]. The device addressed a large barrier in organic optoelectronics, which is the ability to form a high-quality passivation layer on an ultraflexible substrate by making the passivation layers very thin using a low-temperature process. The PLED was constructed using light-emitting polymers and indium tin oxide electrodes. The OPD was constructed with a poly(3-hexylthiophene) (P3HT):(6,6)-phenyl-C61-butyric acid methyl ester (PCBM) active layer, which was manufactured on a 1 μm Parylene substrate, which was used as the passive layer. The lightweight device, which is only 3 μm thick, showed robustness, even under repeated 60% compression. Khan et al. showed a flexible oximeter array in which the active materials for the organic LED (OLED) and the OPD were fabricated on polymer substrates and placed in a grid consisting of photodiodes, red LEDs, and near-infrared LEDS [14]. The device offers a solution to the fundamental problem of only being able to measure PPG signals at a single location by using a reflectance-based array and is therefore capable of measuring blood oxygenation, even in the absence of pulsatile arterial blood signal. The device was able to measure SpO2 with a mean error of 1.1%.

Exciting new optoelectronic research on quantum and nano-based materials is emerging thanks to the ability to tune the performance due to the size of the particles. As shown in Figure 3c, Polat et al. introduced a photodiode made with graphene sensitized with semiconducting quantum dots [58]. Quantum-dot-based graphene photodetectors have high responsivity due to their built-in photoconductive gain. Therefore, the readout electronics can be placed far from the sensor, preserving the form factor of the active sensing area. In addition, the detector’s transparency can be changed by changing the thickness of the quantum-dot layer, which alters the responsivity. This transparent device used ambient light for low power consumption and communicated wirelessly using near-field communication circuitry, as represented by a correlation coefficient of ρ = 0.98, with a state-of-the-art clinical PPG sensor. Kim et al. demonstrated a spirally wrapped CNT-based microelectrode, which is shown in Figure 3d [59]. A CNT-based solution was printed on an agarose hydrogel substrate, where it was then spirally wrapped around a microfiber surface, such as nylon. The CNT electrodes demonstrated a current ratio of ~10^5^ and a maximum field-effect mobility of 0.68 cm^2^ V^−1^ s^−1^, which is comparable to similar flat devices.

For arrhythmia detection, a high signal-to-noise ratio is essential. Thus, the specific design and its optimization are crucial. Pribadi et al. optimized a flexible OLED-OPD patch using an optical simulation [60]. The group optimized the AC/DC ratio of a square-type and cross-type patch. Their results showed that the square-type OPD was the best patch due to the wide area of the OPD, with an OLED drive current between 0.1 and 0.4 mA. The flexibility of the resulting design was 130°, and the heart-rate measurement accuracy was 95%. Khan et al. optimized the geometry of OLEDs and OPDs by designing three geometries: a rectangular geometry, a bracket geometry, and a circular geometry [61]. Both the bracket geometry and the circular geometry showed clear improvement over the rectangular design, where the bracket geometry showed a 39.7% improvement in the red PPG-signal magnitude and an 18.2% improvement in the NIR-channel magnitude, while the circular geometry showed a 48.6% improvement in the red-channel magnitude and a 9.2% improvement in the NI-channel magnitude. These results show promising form-factor and geometry optimization that could increase sensor accuracy and reduce power consumption in future wearable devices.

## 4. Other Signals

### 4.1. Ultrasonic Signals

**Figure 4:**
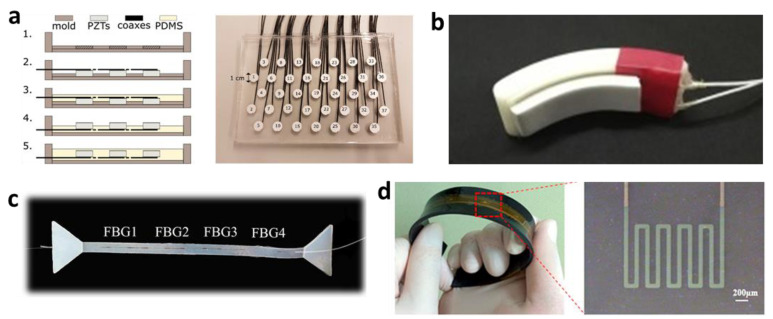
(**a**) Fabrication processes (left) and photo of a fabricated ultrasonic transducer (right). (**b**) Photo of a finger-worn SCG sensor. (**c**) Photo of a skin-like SCG sensor with fibers. (**d**) Flexible strain sensor for heartbeat monitoring.

Doppler ultrasound has been used to track changes in arterial diameter, which can be used to track heart rate. However, commercially available ultrasound monitors are handheld and rigid and therefore not suitable for continuous monitoring. The active layer for ultrasonic transducers is most commonly lead zirconate titanate (PZT) or composites of PZT, as it exhibits high piezoelectric properties and high electromechanical properties [62]. A conformal ultrasonic device was suggested by Wang et al. that could withstand strains of up to 60% [22]. The device utilized a piezoelectric pillar that was hybridized with soft, stretchable components. Liu et al. built on this concept by arranging stretchable ultrasound sensors into a two-dimensional array based on row and column electrodes [63]. The design consisted of PZT blocks, serving as the piezoelectric islands, connected with polyimide (PI) serpentine hinges, allowing for stretchability between the otherwise rigid blocks. Lee et al. showed that a calcium-modified silk could be used as an interface layer between sensor and skin for ultrasound transducer arrays and that it has a similar acoustic impedance to human skin [64]. Hamelmann et al. also introduced an ultrasound array based on PZTs (Figure 4a) [15]. Other materials for stretchable ultrasound substrates include polyethylene naphthalate (PEN), PDMS, acrylic, and PET [60,64,65].

### 4.2. Mechanoelectric Signals

Both ballistocardiography (BCG) and seismocardiography (SCG) have been used to measure cardiac activity based on the heart’s displacement, velocity, and accelerations. BCG measures entire body movement due to cardiac ejection, whereas SCG is a local chest measurement that registers cardiac-induced vibrations. Both are measured in terms of acceleration [65]. Since SCG is typically measured on the body, while BCG is measured using non-contact sensors, SCG has been more commonly used in wearable platforms. While the relationship between SCG and cardiac events is still being studied, the literature has estimated the correlation between certain SCG waves and event timing. For example, a low-frequency acceleration wave can be seen at the start of atrial systole [66]. However, the rigid mismatch between current accelerometers and human skin can introduce whole-body inertia measurements into the SCG signal. Skin-compatible SCG sensors have attempted to address this problem. Ha et al. showed a stretchable e-tattoo SCG based on polyvinylidene fluoride (PVDF) [16]. The sensor also showed a great correlation between the systolic time interval and blood-pressure measurements, and it can simultaneously record ECG signals. Other studies have used PVDF for the SCG sensor while incorporating elements essential for long-term monitoring, like wireless charging and communication (Figure 4b,c) [61,67,68,69]. Wearable phonocardiography (PCG) sensors have also been used to assess the heart. Accelerometer-based mechano-acoustic sensors function similarly to SCG and can pick up auditory frequencies that the human ear cannot hear with a traditional stethoscope. Flexible, wearable stethoscopes based on accelerometry have been developed with flexible substrates and electrodes [67,68]. Kwak et al. also demonstrated a strain-gauge-based heart-rate sensor that can detect not only the timing of the heart pulsation but also the amplitude and shape of the pulse (Figure 4d) [69].

### 4.3. Electrochemical Signals

Metabolic factors, such as glucose level, have been linked to increased risk for arrhythmias. However, monitoring of metabolite levels has traditionally been performed with invasive devices. Recently, wearable glucose-detection devices have allowed for repeated measurement of glucose levels without the burden of an implantable device [70]. Bandodkar et al. reported a tattoo-based noninvasive glucose-monitoring system based on Ag/AgCl ink electrodes and a reagent layer [71]. In vitro characterization proved the system’s ability to detect micromolar levels of glucose, and on-body evaluation proved its ability to detect a rise in glucose after a meal. Sempionatto et al. improved this by creating a stretchable patch for both hemodynamic and metabolic monitoring by combining PZT ultrasound transducers and printed polymer composites [72]. Blood pressure could be monitored through the sensor array, while chemical sensing was realized through sweat detection. This study showed the potential of the integration of acoustic and electrochemical sensing.

## 5. Arrhythmia Detection

Computer-aided ECG and PPG analysis has vastly improved the detection and classification of arrhythmias. The most current process for classification of arrhythmias consists of: (1) pre-processing, where baseline wander and unwanted noises and frequencies are filtered out; (2) feature extraction, where the most important features of a wave are identified, and (3) classification, where the most important features are input into a model to predict the class of arrhythmia of a given signal [73]. For filtering ECG signals, the P- and T-waves are typically found between 0.5 Hz and 10 Hz, while the QRS complex is found between 4 Hz and 20 Hz. Discrete wavelet transforms (DWTs) can be used in combination with low- and high-pass filters to remove unwanted frequencies [74,75]. For PPG signals, wavelet decomposition has also been investigated. However, filtering of motion artifacts for PPG signals has only been proven for weak noise, and very noisy PPG signals need to be discarded [76].

Current arrhythmia-detection models are often based on machine learning, as their accuracy is easily increased with large training datasets. These machine learning methods require feature extraction in either the time domain, frequency domain, time-frequency domain, or nonlinear domain. They are often based on physiological incidents. For example, dimensionality-reduction techniques, such as principal component analysis (PCA), have been used in the time domain, and Fourier transforms have been used in the frequency domain [77,78]. Machine learning models for both ECG and PPG signals have included support vector machine (SVM), multilayer perceptron (MLP), and decision tree (DT) models [78,79,80,81]. The cutting-edge machine learning area is deep learning. Deep learning methods, such as convolutional neural network (CNN), deep belief network (DBN), recurrent neural network (RNN), long short-term memory (LSTM), and gated recurrent unit (GRU), have been applied to arrhythmia detection [82,83,84,85,86]. Table 2 shows these machine learning methods for arrhythmia detection and classification.

Kaisti et al. were able to perfectly distinguish between 13 sinus-rhythm subjects and seven subjects with atrial fibrillation using a k-means clustering-based approach [87]. The input to the algorithm was time-frequency data derived from a soft, band-based MEMS pressure-sensor array, showing the feasibility of combining flexible devices and current arrhythmia detection algorithms to provide high detection accuracy. Improving on this concept, Dong et al. used an arrhythmia-detection system consisting DWT and SVM algorithms, with a novel acetylene carbon black/PDMS ECG recording patch as the input, which achieved a high online classification accuracy of 98.7% [88].

**Table 2:** Comparison of arrhythmia-detection methodologies using wearable devices.

Reference	Device	Target Signal	Arrhythmia Type	Detection Methodology	Accuracy
[12]	iRhythm Zio monitor	ECG	10 types	Deep neural network	ROC = 0.97 F_1_ = 0.837
[89]	Apple watch	PPG, ACC	Atrial fibrillation	Deep neural network	Sens = 0.98 Spec = 0.90
[90]	2-lead Holter monitor	ECG	Atrial fibrillation, atrial flutter, AV junctional rhythm	Hybrid CNN-LSTM	Sens = 0.9787 Spec = 0.9929
[91]	Fingertip pulse oximeter	PPG	Atrial fibrillation	CNN, RNN	AOC = 0.998 AOC = 0.996
[73]	MIT-BIH arrhythmia database	ECG	Ventricular fibrillation	CNN	Acc = 0.9318
[92]	Point-of-care ultrasound	Ultrasound images	Atrial fibrillation	Semi-supervised deep learning network	Acc = 0.79

## 6. Substrate Materials and Skin Interfaces

Due to their flexibility, ease of manufacturing, and low cost, substrates are often made from polymers or fabrics. Elastomers, such as PET, PI, PEN, polyetherimide (PEI), and parylene, are common materials for thin-film-based substrates. Their weak intramolecular forces enable greater elongation and therefore greater stretchability. Substrates such as these offer many opportunities for breakthroughs. For example, Wonryung et al. reported an active, ultra-flexible, multielectrode array that using a 1.2 μm-thick parylene substrate [93]. The 2.6 μm sensor can be used for long-term ECG of dynamically moving hearts due to a 15% strain. Likewise, Shahandashti et al. showed dry stretchable electrodes based on PDMS, which has great biocompatibility, stretchability, and chemical inertness [13]. The substrate showed similar contact impedance to standard wet Ag/AgCl electrodes, though pressure was applied between the electrode and skin using a transparent tape. To improve the adhesion between substrate and skin, Zhang et al. explored a blended film of PEDOT:PSS, waterborne polyurethane, and D-sorbitol prepared by solution processing. These films exhibited low electrode-skin electrical impedances in the frequency range of 1 Hz–10 MHz and adhesion forces above 0.4 N/cm.

## 7. Wearable Devices

Long-term, real-time, continuous monitoring is essential in arrhythmia detection. Thus, advances in form factors and device’s comfort are critical for better wearable devices for successful cardiovascular monitoring. Hardware platforms, such as watches and smartphones, commonly include rigid sensors for measuring ECG and PPG. However, these devices suffer from motion artifacts, data loss, and low accuracy. Recently, form-factor innovations in lightweight wearable devices have increased user comfort and compliance. Breakthroughs in epidermal electronics, for example, have minimized the bulkiness of wearable devices. Wang et al. reported low-cost electronic sensors based on epidermal electronics that minimized motion and sweat artifacts [94]. The sensors reported up to 45% stretchability, adhering to the skin using only van der Waals forces. The 13-μm thick sensor is tape-free and disposable, allowing for ease of both patient compliance and comfort. The durability of wearable devices is also essential in arrhythmia detection. For example, the conductive gels in the traditional Ag/AgCl electrodes degrade over time, resulting in a signal decrease. In addition, the buildup of moisture, dead skin, and material degradation can impact the durability of wearable devices. Xu et al. attempted to mitigate some of these problems through a washable and screen-printed graphene electrode on textiles [95]. The ECG sensor showed negligible change over nine washing cycles and 2000 bending cycles.

## 8. Limitations

Although flexible devices have demonstrated tremendous potential, they are rarely commercialized or used clinically. Low throughput or expensive manufacturing methods make many of these devices difficult to implement widely. For example, non-traditional substrates like PET or TPU cannot be manufactured with large-scale conventional cleanroom fabrication [96,97]. High-throughput manufacturing processes, such as screen printing, have been demonstrated, yet optimal parameters have not yet been discovered. In addition, the resolution of these processes remains low. The elimination of motion artifacts also presents significant challenges. For example, dry electrodes still suffer high impedance with the skin. In addition, powering these devices for long-term monitoring remains a challenge, especially for battery-less devices, and low power consumption is an essential trait for continuous monitoring devices. The addition of wireless transmission also increases the power consumption needed. Bluetooth, for example, consumes up to 5 mW of power, which is more than many thin-film batteries can provide. New machine learning methods can help detect the presence of arrhythmia in heart rates. However, distinguishing between types of arrhythmias and between other classes of heart disease has proven difficult. Machine learning is also limited by the quantity and quality of the training data. In addition, many low-training-data machine learning models are prone to overfitting data and are therefore unable to generalize the testing data. Machine learning is also computationally intensive, making real-time data classification difficult. Finally, the “black-box” nature of machine learning is inherently complex for doctors and clinicians to interpret.

## 9. Conclusions

This review provides many examples to discuss recent advances in arrhythmia detection using flexible and wearable systems that utilize advanced soft materials, flexible designs, and integrated sensors. We summarize the current bioelectric, optoelectric, mechanoelectric, and ultrasonic sensing methods for monitoring various physiological signals related to arrhythmia. These methods are used to detect and classify arrhythmia accurately. We believe that the future of arrhythmia detection lies in further advancements in flexible wearable sensors and automated classification tools using machine learning algorithms. For applications of portable wearable devices in clinical diagnosis, there are areas to improve in terms of materials and sensor performance, such as sensing materials, sensor-to-skin contact quality, impedance control, power-consumption management, miniaturization, wireless data transmissibility, and detection and classification.

## Figures and Tables

**Table 1:** Comparison of flexible devices for heart-rate monitoring.

Reference	Measured Signal	Sensor Location	Substrate Material	Sensor Material	Flexibility
[14]	PPG	Wrist	PEN	PEDOT:PSS	Flexible
[17]	ECG	Arm	unknown	Ag/AgCl	Flexible
[18]	ECG	Chest	PDMS	Carbon black-PDMS nanocomposite	Stretchable
[19]	PPG	Finger	PI	Sb2Se3	Rigid
[20]	ECG	Wrist	Polythiophene	Polyvinyl alcohol/cellulose /PEDOT:PSS	Flexible
[21]	ECG	Forearm	unknown	PEDOT:PSS/WPU/D-sorbitol	Flexible
[22]	Ultrasound	Neck	PI	1–3 Piezoelectric composite	Stretchable
[16]	SCG, ECG	Chest	Tegaderm	PVDF	Stretchable

## Data Availability

No new data were created or analyzed in this study.
